# Growth Environment and Sex Differences in Lipids, Body Shape and Diabetes Risk

**DOI:** 10.1371/journal.pone.0001070

**Published:** 2007-10-24

**Authors:** C. Mary Schooling, Tai Hing Lam, G. Neil Thomas, Benjamin J. Cowling, Michelle Heys, Edward D. Janus, Gabriel M. Leung, for the Hong Kong Cardiovascular Risk Factor Prevalence Study Steering Committee

**Affiliations:** 1 Department of Community Medicine and School of Public Health, The University of Hong Kong, Pokfulam, Hong Kong SAR, China; 2 Clinical Biochemistry Unit, The University of Hong Kong, Pokfulam, Hong Kong SAR, China; 3 Department of Medicine, Western Hospital, Footscray, Victoria, Australia; London School of Hygiene and Tropical Medicine, Peru

## Abstract

**Background:**

Sex differences in lipids and body shape, but not diabetes, increase at puberty. Hong Kong Chinese are mainly first or second generation migrants from China, who have shared an economically developed environment for years, but grew up in very different environments in Hong Kong or contemporaneously undeveloped Guangdong, China. We assessed if environment during growth had sex-specific associations with lipids and body shape, but not diabetes.

**Methodology and Principal Findings:**

We used multivariable regression in a population-based cross-sectional study, undertaken from 1994 to 1996, of 2537 Hong Kong Chinese residents aged 25 to 74 years with clinical measurements of ischaemic heart disease (IHD) risk, including HDL-cholesterol, ApoB, diabetes and obesity. Waist-hip ratio was higher (mean difference 0.01, 95% CI 0.001 to 0.02) in men, who had grown up in an economically developed rather than undeveloped environment, as was apolipoprotein B (0.05 g/L, 95% CI 0.001 to 0.10), adjusted for age, socio-economic status and lifestyle. In contrast, the same comparison was associated in women with lower waist-hip ratio (−0.01, 95% CI −0.001 to −0.02) and higher HDL-cholesterol (0.05 mmol/L, 95% CI 0.0004 to 0.10). The associations in men and women were significantly different (p-values<0.001). There were no such differences for diabetes.

**Conclusions:**

Growth in a developed environment with improved nutrition may promote higher sex-steroids at puberty producing an atherogenic lipid profile and male fat pattern in men but the opposite in women, with tracking of increased male IHD risk into adult life.

## Introduction

Secular trends in ischemic heart disease encompass several features without clear explanation. Firstly, there is a widening of sex difference in premature ischemic heart disease (IHD) with economic development [1], [2], which cannot be fully explained by oestrogen in women [3] or smoking in men [1]. Secondly, the social patterning of ischemic heart disease in men in western populations reversed with economic development over the 20^th^ century, from association with higher to lower social position [4], although this may be the case for women [5]. Mounting evidence from locations outside long-term industrialised countries makes it increasingly clear that the prevalence and social patterning of IHD or its risk factors is not universal but is epidemiologic stage specific [6]–[10]. Finally, in countries with a historically more rapid and recent trajectory of economic development from pre-industrial levels, such as South Korea [11], there is evidence of social inequalities in IHD risk in women but not men [7]. In addition, in long-term industrialised countries with a longer history of economic development there is sometimes a more marked pattern of social inequalities in IHD in women than men [11]–[14], so that the social patterning of IHD risk particularly in men appears to be related to the history and trajectory of socio-economic development.

**Figure 1:**
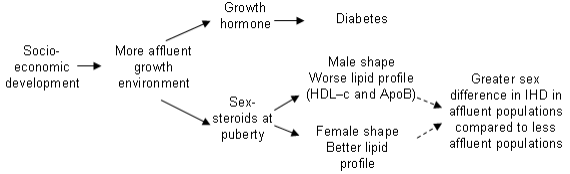
Hypothetical causal pathways.

A (set of ) factor(s) associated with economic development which specifically and universally increased the risk of IHD in men would explain these trends, because it would lead to greater male risk with economic development; it would occur first in the socially advantaged and it would obscure or reduce the generally protective effect of social advantage. In industrialized countries it is well known that a more atherogenic lipid profile and body shape emerges in boys at puberty whilst the reverse happens in girls, due to the action of sex-steroids [15]–[21]. These sex-specific pubertal changes have previously been suggested as contributing to the earlier development of IHD in men [16]. In contrast, although diabetes is a risk factor for IHD, diabetes risk appears to be more strongly related to another hypothalamic-pituitary-endocrine axis, i.e. growth hormone [22] and so should not be affected by pubertal sex-steroids [23]–[27]. With economic development over the last century, there has been little increased rate of diabetes in men compared with women [28]. Figure 1 shows these hypothetical causal pathways. However, it is not known whether these sex-specific pubertal changes are a universal environmentally independent feature of growth and development or an epidemiologically stage specific product of growth in an economically developed environment.

Men and women currently living in Hong Kong share an adult environment, but grew up in very different environments depending on whether they migrated from pre-industrialized Guangdong province or were born in Hong Kong, which was relatively more economically developed than Guangdong province throughout the 20^th^ century.

Most Chinese people in Hong Kong are first or second generation migrants from southern China, mainly from the neighbouring province of Guangdong. The major waves of migration were in the late 1940s, early 1950s and the late 1970s [29]. Migrants were mainly young people looking for work [30], with, until very recently, little migration back to China after 1949. At that time gross domestic product (GDP) per head in China was similar to pre-industrialized western Europe [31]. Guangdong province was an economic backwater until the establishment of the Special Economic Zones near Hong Kong in 1978. In contrast industrialization and economic growth started in the first half of the 20^th^ century in Hong Kong [32]. By the 1930s living conditions in Hong Kong were better than in the neighboring provinces with workers sending remittances back to China [33]. By 1952 per capita GDP was half that of western Europe and by 1995 had overtaken western Europe [31]. We have previously used this natural experiment to examine whether childhood migration was related to self-reports of diabetes, hypertension, hypercholesterolemia or heart disease, obtained from telephone interviews [34]. We previously found no difference in the age-adjusted risk of self-reported IHD (and/or angina) by sex or growth period spent in China or Hong Kong, although self-reported IHD was more common in women than men, suggesting the self-reports were incomplete, thus biasing any relation towards the null. In the present study, we used clinical measurements in a sub-sample to investigate whether the associations of some specific dimensions of IHD risk (body shape and lipid levels) but not others (diabetes) varied by sex according to growth environment location (Guangdong, Hong Kong or both) for Hong Kong residents.

## Materials and Methods

### Sample

The sampling frame and methods of this population-based cross-sectional study in Hong Kong have been described elsewhere in detail [35]. In brief, a probability sample was used to obtain the WHO MONICA [36] recommendation of about 200 participants in each of five 10-year, adult, sex-specific age groups. Randomly generated telephone numbers were used to select households, and within households a Kish grid was used to randomly select respondents. Almost all Hong Kong households (99%) have a telephone [37]. From 1994 to 1996, 7174 Chinese (25–74 years) took part in telephone interviews (a response rate of 78%). The study was supplemented by a relatively small number (556) age 55–74 specifically recruited because there were fewer people of this age in the population. Of the 7730 interviewed, 2900 (38%) came for physical examination. The attendees and non-attendees match the population and non-attendance bias should be small [38]. Appendix S1 shows a comparison of the total sample and those who came for physical examination by socio-demographic characteristics and self-reported health status. For place of birth, exercise, smoking status, self-reported medically diagnosed diabetes or hypertension and general health, the Cohen effect sizes were negligible (<0.1). Effect sizes for job activity, a proxy for socio-economic status, (0.16) and education (0.23) were slightly larger but still acceptable. We excluded pregnant women and those with serious diseases such as cancer and those who were hospitalized. The detailed methods of measurement have been reported [39]. The University of Hong Kong Ethics Committee approved the study and participants gave prior, written, informed consent.

### Exposure assessment

Our primary exposure is growth environment obtained from place of birth, age and length of stay in Hong Kong. Growth environment was categorized as: 1) pre-industrial, i.e. born in Guangdong province and migrated to Hong Kong at age 20 years or older, 2) mixed, i.e. born in Guangdong and migrated to Hong Kong before age 20 years, and 3) more developed, i.e. born and raised in Hong Kong. We chose age 20 years as the cut-off to allow for the completion of possibly slower growth in Guangdong province and to maximise the limited number who migrated as adults given digit preference for migration at age 20 years. We specifically used a separate group for people who migrated before the age of 20, because they experienced potentially detrimental environmental change during growth [40], even though their average experience might be similar to either group. We excluded people born elsewhere to reduce confounding by other unmeasured differences.

### Outcome specification of IHD risk

The main outcome measures were risk factor for IHD in which differences between the sexes become more pronounced at puberty due to the action of sex steroids. At puberty, HDL-cholesterol, body shape and possibly apolipoprotein B (ApoB) change towards a less cardio-protective pattern in males and a more cardio-protective pattern in females [16]–[19], [41]–[43]. Body shape was proxied by waist-hip ratio. HDL-cholesterol, ApoB and waist-hip ratio are all risk factors for IHD, although not necessarily optimal predictors [44]–[47].

A secondary outcome measure was a risk factor for IHD whose precursors also change at puberty but more likely as a function of growth hormone than sex-steroids, i.e. type 2 diabetes [48]–[53]. Diabetes is also a well established risk factor for IHD [44]. Type 2 diabetes was classified using the World Health Organization definition of self-report of previously medically diagnosed diabetes or a fasting plasma glucose ≥7.0 mmol/L or a post-load reading of ≥11.1 mmol/L for those who completed a standard oral glucose tolerance test (consisting of 95% of those not on diabetic medications).

### Statistical analysis

Chi squared tests were used to compare characteristics between groups. Locally weighted regression and scatter plot smoothing (LOWESS) was used to examine risk factors across the age-range. The association of each risk factor with growth environment adjusted for potential confounding factors was assessed in men and women separately using multiple regression. Mean differences and 95% confidence intervals (CIs) were calculated from linear regression for continuous outcomes. Odds ratios and 95% CIs were calculated from logistic regression for diabetes. Whether growth environment had a different association in men and women was assessed from the heterogeneity across strata and the significance of interaction terms. We tested for interactions by running models with and without the interaction term and examining the statistical significance of the likelihood ratio test of the difference between the two models on the relevant chi-squared distribution. A two-tailed p value of less than 5% was considered statistically significant.

Potential confounding factors considered were education, type of housing, use of alcohol, smoking and exercise categorized as in Table 1 and age. First we adjusted for age (model 1). Next, we added all other confounders (model 2). Finally, we also adjusted HDL-cholesterol, ApoB and diabetes for waist-hip ratio and body mass index (model 3). Each analysis used all relevant complete data (available for 97%).

**Table 1:** Characteristics by sex and growth environment for 2357 Hong Kong residents born in Guangdong province (China) or Hong Kong and examined in 1994 to 1996

	Male (N = 1246)				Female (N = 1291)			
	Growth environment				Growth environment			
	Pre-industrial*	Mixed †	More developed ‡	χ^2 ^p-value	Pre-industrial*	Mixed †	More developed ‡	χ^2 ^p-value
Education (n)	245	248	750		234	219	831	
Primary or less (%)	45.7	46.4	16.0		62.4	60.7	29.6	
Secondary (%)	42.9	42.3	56.5		33.3	37.0	52.8	
Matriculation or above (%)	11.4	11.3	27.5	<0.001	4.3	2.3	17.6	<0.001
Type of housing (n)	247	248	748		233	220	832	
Private or home owner (%)	54.7	50	41.4		48.1	44.1	41.1	
Public and other (%)	45.3	50	58.6	<0.001	51.9	55.9	58.9	0.15
Ever use of alcohol (n)	247	247	751			218	834	
Yes (%)	53.0	57.9	56.9		12.0	22.9	21.2	
No (%)	47.0	42.1	43.1	0.49	88.0	77.1	78.9	0.003
Smoking status (n)	247	247	750			220	833	
Never (%)	38.9	44.9	59.1		96.6	97.7	94.0	
Ex-smoker (%)	20.6	15.4	8.3		0.4	0.0	0.8	
Current smoker (%)	40.5	39.7	32.7	<0.001	3.0	2.3	5.2	0.14
Leisure exercise in past month (n)	247	248	747			218	833	
Yes (%)	37.7	42.3	50.2		39.9	37.6	43.2	
No (%)	62.3	57.7	49.8	0.001	60.1	62.4	56.8	0.27
Self rated general health (n)	247	247	750			219	833	
Very good/good (%)	92.7	96.4	98.3		89.3	86.8	96.7	
Poor/very poor (%)	7.3	3.6	1.7	<0.001	10.7	13.2	4.3	0.27

* Born in Guangdong province and lived there until 20 years of age or older

† Born in Guangdong province and migrated to Hong Kong before the age of 20 years

‡ Born in Hong Kong

## Results

Of the 2900 participants, 5 were excluded because they were pregnant, 1 was excluded because place of birth was unknown, 350 were excluded because of birth outside Guangdong or Hong Kong, and 7 migrants from Guangdong were excluded because their duration of residence in Hong Kong was unknown. Of the remaining 2537, 1586 were Hong Kong born and 951 were migrants from Guangdong (Table 2). Just over half the migrants (482) had grown up in Guangdong and mostly migrated to Hong Kong in their 20s (72%) and had lived in Hong Kong for many years; mean 28.7 years in men and 26.9 years in women. The other 469 had migrated from Guangdong to Hong Kong before the age of 20 years.

**Table 2:** Place of birth, migration status and growth environment by age and sex for 2895 Hong Kong residents examined in 1994 to 1996

Place of birth	Place of birth	Growth environment	Men				Women				Total
			Age group in years			all	Age group in years			All	
			25–39	40–59	60–74		25–39	40–59	60–74		
Hong Kong											
	Hong Kong born	More developed	425	268	58	751	435	335	65	835	1586
Guangdong province in mainland China			62	231	204	497	69	223	169	461	958
	Child migrant from Guangdong (at age <20 years)	Mixed	36	124	88	248	32	129	60	221	469
	Adult migrant from Guangdong (at age ≥20 years)	Pre-industrial	26	107	114	247	36	92	107	235	482
	Unknown age of migration		0	0	2	2	1	2	2	5	7
Other provinces in mainland China		n/a	16	55	42	113	20	67	26	113	226
	Child migrant (moved at age <20 years)		10	18	13	41	8	16	5	29	70
	Adult migrant (moved at age 20 years or older)		6	36	29	71	12	50	21	83	154
	Unknown age at migration		0	1	0	1	0	1	0	1	2
Outside mainland China and Hong Kong		n/a	8	31	12	51	14	45	14	73	124
Unknown place of birth		n/a				0			1	1	1
Total			511	585	316	1412	538	670	275	1483	2895

People born and raised in Hong Kong were more likely to have attained higher education than the other two groups (Table 1). In contrast men from Guangdong were more likely to smoke, take no exercise and report poorer self-rated health than the other groups and these associations remained after adjustment for age (data not shown). There was no use of lipid-lowering medication.

**Figure 2:**
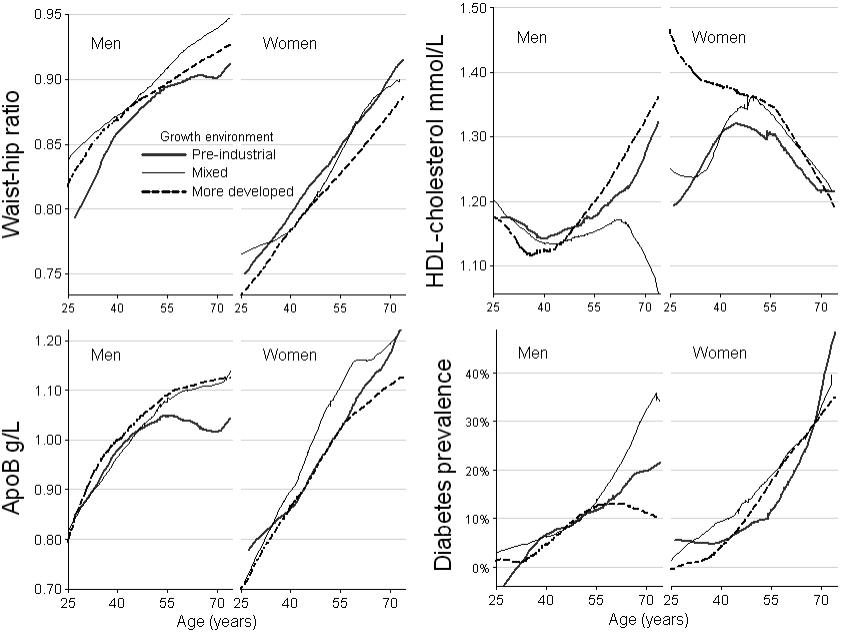
Relation of waist-hip ratio, HDL-cholesterol, ApoB and diabetes prevalence (%) with age using LOWESS plots by growth environment in men and women for 2357 Hong Kong residents born in Guangdong province (China) or Hong Kong and examined in 1994 to 1996.

Differences by growth environment in waist-hip ratio and ApoB in men and women were present across the age-range, although less clearly so for HDL-cholesterol particularly in men (Figure 2). However there were no obvious differences for diabetes.

Waist-hip ratio and ApoB were higher in men who had grown up in Hong Kong than in men who had grown up Guangdong (Table 3), adjusted for age (model 1) or additionally adjusted for socio-economic status and lifestyle (model 2). In contrast, waist-hip ratio was lower and HDL-cholesterol was higher in women who had grown up in Hong Kong than in women who had grown up in Guangdong, adjusted for age (model 1) or additionally adjusted for socio-economic status and lifestyle (model 2). These different associations with waist-hip ratio, HDL-cholesterol and ApoB for men and women by growth environment were significant. Further adjustment of HDL-cholesterol and ApoB for waist-hip ratio and body mass index (model 3) attenuated the associations.

**Table 3:** Mean values and adjusted differences with 95% confidence intervals in waist-hip ratio, HDL-cholesterol and ApoB by growth environment in men and women for 2357 Hong Kong residents born in Guangdong province (China) or Hong Kong and examined in 1994 to 1996

				Men					Sex difference p-value	Women				
	Mean (SD)			Growth environment						Growth environment				
	Men	Women	Model	n	Pre-industrial*	Mixed †	More developed ‡	trend p-value		n	Pre-industrial *	Mixed †	More developed ‡	trend p-value
Waist-hip	0.88	0.80	1	1244	reference	0.02	0.01	0.10	<0.001	1290	reference	−0.01	−0.02	0.003
ratio	(0.07)	(0.08)				0.01 to 0.03	0.001 to 0.02					−0.02 to 0.005	−0.03 to −0.005	
			2	1232	reference	0.02	0.01	0.08	<0.001	1266	reference	−0.01	−0.01	0.03
						0.01 to 0.03	0.001 to 0.02					−0.02 to 0.004	−0.02 to −0.001	
HDL	1.16	1.35	1	1243	reference	−0.03	−0.01	0.88	<0.001	1287	reference	0.03	0.06	0.02
(mmol/L)	(0.30)	(0.32)				−0.09 to 0.02	−0.06 to 0.04					−0.03 to 0.09	0.01 to 0.11	
			2	1231	reference	−0.04	−0.01	0.70	<0.001	1263	reference	0.03	0.05	0.05
						−0.09 to 0.01	−0.06 to 0.03					−0.03 to 0.09	0.0004 to 0.10	
			3	1229	reference	−0.01	0.01	0.59	0.04	1260	reference	0.02	0.04	0.08
						−0.06 to 0.04	−0.03 to 0.06					−0.04 to 0.08	−0.01 to 0.09	
ApoB	1.00	0.92	1	1118	reference	0.04	0.05	0.048	<0.001	1144	reference	0.06	−0.01	0.34
(g/L)	(0.27)	(0.32)				−0.01 to 0.09	0.002 to 0.09					0.001 to 0.12	−0.06 to 0.04	
			2	1107	reference	0.04	0.05	0.051	<0.001	1125	reference	0.06	−0.01	0.41
						−0.01 to 0.09	0.001 to 0.10					−0.003 to 0.12	−0.06 to 0.04	
			3	1105	reference	0.02	0.04	0.11	<0.001	1122	reference	0.06	−0.00	0.55
						−0.03 to 0.07	−0.01 to 0.08					0.01 to 0.12	−0.05 to 0.05	

Model 1 adjusted for age

Model 2 adjusted for age, education, housing, smoking status, alcohol use status and leisure exercise

Model 3 adjusted for age, education, housing, smoking status, alcohol use status, leisure exercise, waist-hip ratio and body mass index

* Born in Guangdong province and lived there until 20 years of age or older

† Born in Guangdong province and migrated to Hong Kong before the age of 20 years

‡ Born in Hong Kong

Diabetes risk was similar for men and women who grew up in Hong Kong or Guangdong (Table 4) in all three models, although men and women who had experienced environmental change during growth tended to have a higher risk of diabetes. There was no evidence that the association of diabetes risk with growth environment varied between men and women. This finding was not due to lack of power due to using a dichotomous outcome, as there was also no evidence of different associations with fasting plasma glucose or post-load plasma glucose by growth environment in men and women (interaction p-values 0.28 and 0.41 respectively in model 2).

**Table 4:** Prevalence and adjusted odds ratio with 95% confidence intervals of diabetes by growth environment in men and women for 2357 Hong Kong residents born in Guangdong province (China) or Hong Kong and examined in 1994 to 1996.

			Men						Sex difference p-value	Women					
Prevalence			Growth environment							Growth environment					
Men	Women	Model	n	Pre-industrial *	Mixed †	More developed ‡	trend p-value	Hosmer-Lemeshow p-value		n	Pre-industrial *	Mixed †	More developed ‡	Trend p-value	Hosmer-Lemeshow p-value
8.4%	9.5%	1	1245	1	1.33	0.88	0.66	0.15	0.88	1289	1	1.58	1.07	0.90	0.87
					0.78 to 2.27	0.50 to 1.55						0.93 to 2.69	0.63 to 1.81		
		2	1233	1	1.32	0.90	0.72	0.52	0.78	1265	1	1.56	1.09	0.82	0.72
					0.77 to 2.26	0.51 to 1.59						0.91 to 2.67	0.64 to 1.85		
		3	1231	1	1.01	0.74	0.31	0.51	0.45	1262	1	1.72	1.16	0.66	0.10
					0.57 to 1.77	0.41 to 1.33						0.98 to 3.01	0.67 to 1.99		

Model 1 adjusted for age

Model 2 adjusted for age, education, housing, smoking status, alcohol use status and leisure exercise

Model 3 adjusted for age, education, housing, smoking status, alcohol use status, leisure exercise, waist-hip ratio and body mass index

* Born in Guangdong province and lived there until 20 years of age or older

† Born in Guangdong province and migrated to Hong Kong before the age of 20 years

‡ Born in Hong Kong

## Discussion

Taking advantage of a unique natural experiment generated by migration, we found that different environments during growth were associated differently in men and women with the IHD risk factors affected by sex-steroids at puberty. Men, who had grown up in a relatively more economically developed environment (i.e. Hong Kong) had a more male shape and less favourable lipids than men who had grown up in a pre-industrial environment (economically undeveloped Guangdong). In contrast, women who had grown up in a relatively more economically developed environment had a more feminine shape and more favourable lipids than women who had grown up in a pre-industrial environment. These findings on lipids were attenuated slightly by adjustment for obesity, as wait hip ratio and body mass index suggesting there may be some underlying commonality. However, growth environment was not related to diabetes in men or women, although environmental change during growth to a more developed environment was associated with a non-significantly higher risk of diabetes.

To our knowledge, these findings are unique. There are few other ethnically homogeneous, developed populations, whose adult environments are similar, but whose growth environments were radically different. Nor, have male-female differences in IHD risk factors specifically been reported in such a setting, although there are some similar male-female differences in the long-standing Japanese migrant community in Brazil [54]. However, our findings are consistent with the ecological observation that a female preponderance in obesity disappears with economic development [55]. Moreover, our specific observation that growth environment can have different associations in men and women with HDL-cholesterol and waist circumference but not a pre-cursor of diabetes (higher Hba1c) has previously been demonstrated, but not explained, in the United Kingdom [13]. On the other hand our findings are not consistent with IHD or its risk factors being associated with poor childhood conditions [56], [57]. However, almost all such studies come from long-term industrialized countries where the reversal of the social patterning of ischemic heart disease in men has already taken place, and the gradation between levels of childhood conditions is not comparable with the difference between pre-industrial conditions in Guangdong and economically developed Hong Kong. There is one case-control study from Hong Kong where acute myocardial infarction had little association with education or childhood crowding, but was associated with a non-working mother, infrequent meat consumption as a child and inadequate food intake as a child [58]. These inconsistent findings are difficult to interpret as most cases did not answer the questions on childhood diet [59]. On the other hand, that higher levels of some risk factors were seen in the group who migrated from a pre-industrial environment to a relatively more developed environment during growth, might be consistent with accelerated or ‘catch-up’ growth increasing cardiovascular risk [60], [61]. However there is little evidence that accelerated growth has different associations by sex, or is specific to lipids and body shape but not diabetes. Moreover, the biological mechanisms by which accelerated growth increases risk are not clearly understood.

Given the nature of our study there are many limitations and provisos. We took advantage of an uncontrolled natural experiment, so we are not showing precise point estimates of differences in lipids and body shape in men and women by growth environment. Economic differences between Guangdong and Hong Kong were possibly smallest during the early 20^th^ century and greatest after 1945; nevertheless there was probably always a relative difference in favour of Hong Kong. Moreover, we found reasonably consistent differences across the age range (Figure 2). The participants may not have been completely representative; they may be better educated, however adjustment for education (model 2 compared with model 1) made little change to the estimates suggesting that education was not a major confounder. The long life expectancy and low rate of cardiovascular disease in Hong Kong make it unlikely that the findings result from premature adult deaths; after about 8 years of follow-up 29 of the 2900 participants had died from cardiovascular disease.

Total life exposure rather than exposures during growth may be the crucial factor; however we essentially compared men and women with similar total life exposures and found different associations by growth environment. Dietary data is not available for all these participants, so we cannot exclude the possibility that migrant men retained a diet specifically protective for obesity and lipids, but not diabetes, whilst the migrant women did not, although it is not easy to envisage such a diet. None of the participants reported using lipid lowering drugs, which makes treatment differences by sex and place of origin unlikely. In addition, the migrant men were more likely to smoke and to report poor self-rated health than Hong Kong born men. Moreover, the specificity of these findings to body shape and lipids but not diabetes makes it unlikely that they are the result of either different lifestyles by sex and place of origin, differential residual confounding in men and women or self-selection of healthy migrant men. Finally, only 38% of those originally surveyed attended the clinic for physical examination. Our findings would be biased if, in those from the original sample who did not attend for examination, there were systematically different associations of sex and growth environment to lipids, body shape or diabetes. Comparing the attenders with the non-attenders we found little evidence that the associations of sex and growth environment to socio-demographic characteristics or health varied with attendance status (Appendix S1).

Notwithstanding these provisos our study provides some aetiological evidence that increased risk of IHD in men compared to women may be related to growing up in a more economically developed environment. There is a plausible biological mechanism by which the dramatically improved living conditions associated with the epidemiological transition could have opposite effects on lipids and body shape in men and women, without causing sex differences in diabetes risk. Cholesterol from the diet or other sources is needed for the synthesis of sex-steroids. Animal experiments show that under-feeding reduces testosterone [62]–[64] and oestrogen levels [65] at puberty. Even in universally well-fed human populations, slight changes in diet affect sex-steroids in girls [66] although not in boys [67], where larger nutritional differences are needed [68]. Sex steroids are responsible for the sexual dimorphism in body shape and lipids that arises at puberty [17], [18], [69]. However, changes in pre-cursors of diabetes at puberty appear to be more strongly related to another hypothalamic-pituitary-endocrine axis, i.e. growth hormone [70]–[75]. Lipids and central obesity track across the life course from adolescence [76]. We speculate that nutritionally driven increases in sex-steroids at puberty, manifested by earlier and more intense puberty seen in economically developed environments [77] but not in very poor environments [68], permanently increase sexual dimorphism in shape, HDL-cholesterol and ApoB with correspondingly increased IHD risk in men compared to women.

These findings may be consistent with a biologically plausible hypothesis, but they are only very preliminary, and require confirmation, because there are potential implications for the large proportion of the global population currently undergoing rapid economic development. Our hypothesis implies that transition to a high fat diet at or before puberty could generate a subsequent epidemic of heart disease in men and that growing boys should perhaps be a target for intervention. On the other hand in girls the potentially cardio-protective effects of the nutritional transition need to be weighed against potentially greater risk of other disease in later life.

### Conclusion

Rather than being entirely an intrinsic sex-specific property, increased risk of IHD in men compared to women may be associated with growing up in an economically developed environment, thus providing a socio-biological explanation for the secular trends and sex differences in IHD seen with economic development. In considering the developmental origins of health, puberty, as well as fetal and early life, should be considered as a key developmental window, with potentially life-long effects on health. Understanding the long-term impacts of different patterns of pubertal development are of key relevance to the large proportion of the global population currently under-going epidemiological transition with rapidly changing patterns of pubertal development.

## Supporting Information

Appendix S1 (0.15 MB DOC)
