# Supplementary material for: Growth Environment and Sex Differences in Lipids, Body Shape and Diabetes Risk
Source: PLoS One. 2007 Oct 24;2(10):e1070. doi: 10.1371/journal.pone.0001070 (PMC2031823; doi:10.1371/journal.pone.0001070)
Supplement: Appendix S1 — (0.15 MB DOC) [file pone.0001070.s001.doc]

Appendix S1. Characteristics by attendance at physical examination of 7730 Hong Kong residents in the original sample interviewed over the telephone in 1994 to 1996

|  | | |  | Attended for physical examination | | | | | Age and sex adjusted odds ratio of attending | | p-value for interaction of attendance status with | | |  |
| --- | --- | --- | --- | --- | --- | --- | --- | --- | --- | --- | --- | --- | --- | --- |
|  | | |  | No | | Yes | | p-value* | OR | 95% CI | sex | Growth environ-ment | Sex and growth environ-ment |  |
|  | | |  | n | % | n | % |  |  |  |
| N | | |  |  |  |  |  |  |  |  |  | | |
| Age: mean and ±SD | | | | 47.5 | 14.8 | 45.8 | 12.9 | <0.01 |  |  |  |  |  |  |
| Sex | | | |  |  |  |  |  |  |  |  |  |  |  |
|  | Men | | | 2362 | 48.9 | 1412 | 48.7 |  | 1 |  |  |  |  |  |
|  | Women | | | 2468 | 51.1 | 1488 | 51.3 | 0.86 | 1.01 | 0.92 to 1.11 | n/a | 0.47 | n/a |  |
| Education | | | |  |  |  |  |  |  |  |  |  |  |  |
|  | Primary or less | | | 2129 | 44.4 | 977 | 33.8 |  | 1 |  |  |  |  |  |
|  | Secondary | | | 2052 | 42.8 | 1396 | 48.3 |  | 1.49 | 1.32 to 1.67 |  |  |  |  |
|  | Matriculation or above | | | 616 | 12.8 | 517 | 17.9 | <0.01 | 1.84 | 1.58 to 2.15 | 0.31 | 0.12 | 0.09 |  |
| Housing | | | |  |  |  |  |  |  |  |  |  |  |  |
|  | Private or home owner | | | 2464 | 51.8 | 1239 | 42.9 |  | 1 |  |  |  |  |  |
|  | Public and other | | | 2296 | 48.2 | 1651 | 57.1 | <0.01 | 1.40 | 1.28 to 1.54 | 0.78 | 0.29 | 0.73 |  |
| Physical activity in job | | | |  |  |  |  |  |  |  |  |  |  |  |
|  | No job | | | 1777 | 36.8 | 850 | 29.3 |  | 1 |  |  |  |  |  |
|  | Sitting job | | | 637 | 13.2 | 453 | 15.6 |  | 1.46 | 1.25 to 1.71 |  |  |  |  |
|  | Mild activity job | | | 982 | 20.3 | 674 | 23.4 |  | 1.43 | 1.24 to 1.64 |  |  |  |  |
|  | Moderate/heavy activity job | | | 1434 | 29.6 | 922 | 31.8 | <0.01 | 1.41 | 1.23 to 1.61 | 0.18 | 0.56 | 0.36 |  |
| Place of birth | | | |  |  |  |  |  |  |  |  |  |  |  |
|  | Hong Kong | | | 2425 | 50.3 | 1590 | 54.9 |  | 1 |  |  |  |  |  |
|  | Other | | | 2398 | 49.7 | 1309 | 45.2 | <0.01 | 0.92 | 0.83 to 1.02 | 0.29 | n/a | n/a |  |
| Growth environment | | | |  |  |  |  |  |  |  |  |  |  |  |
|  | Guangdong | | | 1120 | 26.1 | 483 | 19.0 |  | 1 |  |  |  |  |  |
|  | Mixed | | | 739 | 17.3 | 469 | 18.5 |  | 1.43 | 1.22 to 1.67 |  |  |  |  |
|  | Hong Kong | | | 2425 | 56.6 | 1590 | 62.6 | <0.01 | 1.35 | 1.17 to 1.55 | 0.47 | n/a | n/a |  |
| Ever use of alcohol | | | |  |  |  |  |  |  |  |  |  |  |  |
|  | | Yes | | 1443 | 30.0 | 1068 | 36.9 |  | 1 |  |  |  |  |  |
|  | | No | | 3371 | 70.0 | 1826 | 63.1 | <0.01 | 0.70 | 0.63 to 0.78 | 0.01 | 0.52 | 0.27 |  |
| Smoking status | | | |  |  |  |  |  |  |  |  |  |  |  |
|  | Never | | | 3451 | 71.6 | 2154 | 74.4 |  | 1 |  |  |  |  |  |
|  | Ex-smoker | | | 300 | 6.2 | 185 | 6.4 |  | 1.06 | 0.86 to 1.30 |  |  |  |  |
|  | Current smoker | | | 1070 | 22.2 | 555 | 19.2 | <0.01 | 0.81 | 0.71 to 0.93 | 0.01 | 0.67 | 0.09 |  |
| Leisure exercise in past month | | | |  |  |  |  |  |  |  |  |  |  |  |
|  | | Yes | | 1888 | 39.3 | 1235 | 42.8 |  | 1 |  |  |  |  |  |
|  | | No | | 2921 | 60.7 | 1652 | 57.2 | <0.01 | 0.88 | 0.80 to 0.96 | 0.48 | 0.54 | 0.90 |  |
| Self rated general health | | | |  |  |  |  |  |  |  |  |  |  |  |
|  | | Very good/good | | 4460 | 92.6 | 2720 | 94.0 |  | 1 |  |  |  |  |  |
|  | | Poor/very poor | | 359 | 7.5 | 173 | 6.0 | 0.01 | 0.86 | 0.71 to 1.05 | 0.01 | 0.45 | 0.052 |  |
| Self-reported diabetes | | | |  |  |  |  |  |  |  |  |  |  |  |
|  | | Yes | | 246 | 5.1 | 109 | 3.8 |  | 1 |  |  |  |  |  |
|  | | No | | 4576 | 94.9 | 2788 | 96.2 | <0.01 | 1.22 | 0.96 to 1.55 | 0.78 | 0.77 | 0.91 |  |
| Angina or self-reported CHD | | | |  |  |  |  |  |  |  |  |  |  |  |
|  | | Yes | | 237 | 4.9 | 158 | 5.5 |  | 1 |  |  |  |  |  |
|  | | No | | 4565 | 95.1 | 2717 | 94.5 | 0.28 | 0.83 | 0.67 to 1.03 | 0.60 | 0.25 | 0.64 |  |
| Self-reported hypertension | | | |  |  |  |  |  |  |  |  |  |  |  |
|  | | Yes | | 602 | 12.5 | 316 | 10.9 |  | 1 |  |  |  |  |  |
|  | | No | | 4222 | 87.5 | 2584 | 89.1 | 0.04 | 1.04 | 0.89 to 1.21 | 0.84 | 0.57 | 0.64 |  |
| Self reported hypercholestrima | | | |  |  |  |  |  |  |  |  |  |  |  |
|  | | Yes | | 259 | 5.4 | 218 | 7.5 |  | 1 |  |  |  |  |  |
|  | | No | | 4561 | 94.6 | 2681 | 92.5 | <0.01 | 0.63 | 0.52 to 0.77 | 0.46 | 0.92 | 0.37 |  |

* oneway ANOVA p-value for age and χ^2^ p-value for all other variables
